# A New Strategy for Targeting UCP2 to Modulate Glycolytic Reprogramming as a Treatment for Sepsis A New Strategy for Targeting UCP2

**DOI:** 10.1007/s10753-024-01998-4

**Published:** 2024-03-02

**Authors:** Na Li, Jiali Deng, Junli Zhang, Fei Yu, Fanghang Ye, Liyuan Hao, Shenghao Li, Xiaoyu Hu

**Affiliations:** 1 Department of Infectious Diseases, Hospital of Chengdu University of Traditional Chinese Medicine, Chengdu, China; 2 Department of Clinical Medicine, Chengdu University of Traditional Chinese Medicine, Chengdu, China; 3 Jiangsu Provincial Hospital of Traditional Chinese Medicine, Nanjing, China

**Keywords:** UCP2 overexpression, Glycolysis, Excessive inflammation, Immune suppression, Sepsis

## Abstract

Sepsis is a severe and life-threatening disease caused by infection, characterized by a dysregulated immune response. Unfortunately, effective treatment strategies for sepsis are still lacking. The intricate interplay between metabolism and the immune system limits the treatment options for sepsis. During sepsis, there is a profound shift in cellular energy metabolism, which triggers a metabolic reprogramming of immune cells. This metabolic alteration impairs immune responses, giving rise to excessive inflammation and immune suppression. Recent research has demonstrated that UCP2 not only serves as a critical target in sepsis but also functions as a key metabolic switch involved in immune cell-mediated inflammatory responses. However, the regulatory mechanisms underlying this modulation are complex. This article focuses on UCP2 as a target and discusses metabolic reprogramming during sepsis and the complex regulatory mechanisms between different stages of inflammation. Our research indicates that overexpression of UCP2 reduces the Warburg effect, restores mitochondrial function, and improves the prognosis of sepsis. This discovery aims to provide a promising approach to address the significant challenges associated with metabolic dysfunction and immune paralysis.

## INTRODUCTION

Sepsis is a life-threatening organ dysfunction caused by dysregulation of the body’s immune response to infection [1], which can be severe enough to lead to MODS and septic shock, or even death. Based on a comprehensive global study on sepsis epidemiology, it is estimated that there are nearly 500,000 cases of sepsis annually worldwide, making up around 20% of all global fatalities. Sepsis is recognized as a significant contributor to the global health and economic burden, with variations in its incidence and mortality rates across different regions [2], Generally, low- and middle-income countries tend to experience higher rates of sepsis than higher-income countries [3, 4]. The typical clinical manifestations of sepsis include fever, tachycardia, leukocytosis, hypotension, coagulation dysfunction, and altered mental status [5], with fever being the most common symptom [6]. The management of sepsis primarily involves controlling the infection, maintaining hemodynamic stability, and modulating the host response [7]. Although the survival rate of sepsis has improved over the past 40 years [8], however, current clinical treatment still primarily relies on early recognition and commonly adopts treatment strategies such as fluid resuscitation, antimicrobial therapy, and symptomatic treatment [9]. However, clinical efficacy is limited, and its treatment is time-dependent [10]. Without timely and effective intervention, sepsis can progress to septic shock, with a mortality rate of around 38% [11]. While significant progress has been made in genomics, transcriptomics, proteomics, and metabolomics research, specific treatment modalities or highly effective drugs for sepsis have not yet been developed [12].

Sepsis is a multifactorial syndrome, and its complex pathogenesis limits the development of treatment strategies. The prevailing understanding of its pathogenesis encompasses mitochondrial damage [13], systemic inflammatory network effects [14], immune dysfunction [15], and other complications. Mitochondria are essential for living organisms and participate in various pathological and physiological processes within the body [16]. Their function is affected by the inflammatory response [17] and is a significant factor contributing to poor prognosis in sepsis [18]. Mitochondrial dysfunction manifests in various aspects, such as excessive production of reactive oxygen species (ROS), depletion of ATP, structural changes in mitochondria, and increased cellular apoptosis [19]. Uncoupling protein 2 (UCP2) is an anion transporter protein that is predominantly found on the inner membrane of mitochondria. It is widely expressed in various tissues and organs throughout the body. It plays a crucial role in multiple pathophysiological conditions within mitochondria [20]. During sepsis, there is conflicting evidence regarding the upregulation or downregulation of UCP2 in different tissues and the resulting outcomes.

Sepsis patients often exhibit a “dysregulated inflammatory response”: during the early stages of sepsis, activated immune cells initiate innate immunity, resulting in a significant increase in the inflammatory response aimed at clearing invading pathogens within the host. If the initial response is not timely and effectively controlled, the excessive inflammatory response will damage tissue and organs, subsequently leading to a more sustained immune suppression [21]. During this stage, the function of immune cells is impaired, and the body is unable to mount a response to secondary infections, which is a key factor leading to secondary infections and the development of multiple organ dysfunction syndrome (MODS) [22]. The inflammatory response and immune suppression typically occur sequentially or simultaneously [23]. In order to meet the body’s energy demands, immune cells in the excessive inflammatory stage often exhibit a metabolic shift from oxidative phosphorylation (OXPHOS) to aerobic glycolysis [24], similar to the Warburg effect proposed by Otto Warburg [25] in 1920 in tumor cells. This means that immune cells rely on glycolysis to generate adenosine triphosphate (ATP), and mitochondrial dysfunction is the underlying cause of this effect [26]. Reversing the Warburg effect has been widely applied as a therapeutic strategy in tumor cells [27]. Bar-Or et al. [28] have suggested that reversing the Warburg effect may lead to better clinical outcomes for sepsis patients. UCP2 has been considered a molecular basis for the Warburg effect [29].

UCP2 not only serves as a key mediator in the pathogenesis of sepsis [30], but it also acts as a “metabolic switch” connecting glucose oxidation and mitochondrial metabolism, promoting the oxidation of glutamine and fatty acids instead of glucose-derived pyruvate oxidation [31]. Additionally, UCP2 reduces the production of reactive oxygen species (ROS) [32]. When UCP2 is overexpressed, it generates retrograde signals from the mitochondria, which in turn modifies the expression of glycolytic and oxidative enzymes, leading to enhanced oxidative phosphorylation. These findings support the idea that UCP2 overexpression can shift cellular metabolism from glycolysis to oxidative phosphorylation, with UCP2 exerting its effects by controlling mitochondrial substrates rather than acting as a membrane potential uncoupling protein [33].

Developing targeted strategies to modulate UCP2 for the treatment of sepsis is undoubtedly a therapeutic approach with great potential, based on the following rationale: (1) during sepsis, differential expression of UCP2 leads to distinct and even opposing biological effects. For instance, studies conducted by Ding et al. [34] have discovered that while UCP2 expression is increased in septic mice, UCP2 overexpression can actually mitigate mitochondrial dysfunction and reduce the expression of inflammatory factors. During sepsis, the immune status of patients significantly influences their prognosis. UCP2 is expressed in various immune cells, including macrophages [35] and mast cells [36], and plays a crucial role in immune responses [37]. During the peak of inflammation in sepsis, immune cells and organ cells prioritize glycolysis as their main source of energy, while OXPHOS is inhibited [38]. Glycolysis can rapidly provide energy for the body’s needs in the early stages, playing a critical role in host defense and inflammation [39]. However, the production of high levels of lactate by glycolysis can promote immune cell death or inactivation, exacerbating immune suppression and disrupting the body’s immune homeostasis [40]. In the late stage of sepsis, mitochondrial dysfunction leads to the inability to restore OXPHOS, resulting in sustained organ damage [41]. UCP2 plays a positive role during sepsis [42], regulating the shift from OXPHOS to glycolysis and inhibiting damage caused by high levels of glycolysis. Inhibiting the expression of UCP2 promotes this effect, while overexpression of UCP2 can weaken the Warburg effect [33], which is beneficial for the recovery of mitochondrial respiration and function [43].

Based on the above, it has been found that UCP2 is involved in both metabolic and immune processes. As a result, it has been identified as a promising candidate for intervening in the occurrence, progression, and prognosis of sepsis. This review aims to elaborate on the potential therapeutic effect of overexpressing UCP2 on sepsis from the perspective of metabolic and immune regulation. This central mechanism underlying the therapeutic effect may be based on metabolic reprogramming, which is a complex process that aims to address the intricate relationship between metabolism and immunity during sepsis, and providing possible therapeutic strategies for the treatment of sepsis.

## UCP2 AND SEPSIS

### Physiology and Biochemistry of UCP2

Although many studies have confirmed the participation of UCP2 in multiple physiological and pathological processes, its precise biological functions are still a matter of controversy. Therefore, we will first start with the physiological and biochemical aspects of UCP2 to better understand its role in sepsis.

#### The Physiological and Pathological of UCP2

Mitochondria play a critical role in key cellular processes such as metabolism, the generation of reactive oxygen species (ROS), and programmed cell death. They primarily convert energy into ATP through coupling between the electron transport chain and oxidative phosphorylation. Uncoupling proteins (UCPs) are located on the inner membrane of mitochondria and belong to the SLC25 transporter protein family [44]. They serve as mitochondrial carriers that act as protectors against reactive oxygen species and regulators of ATP-dependent processes [45]. Consisting of at least five protein subtypes, UCP1-5 have garnered significant attention from researchers as potential therapeutic targets for various diseases [46]. UCPs exhibit tissue-specific expression patterns. UCP1 is specifically expressed in brown adipose tissue [47]. UCP2 is widely expressed in various tissues, including the spleen, lungs, stomach, and white adipose tissue [48]. UCP3 is predominantly expressed in skeletal muscle [45], while UCP4 and UCP5 are primarily expressed in the central nervous system (CNS) [49].

UCP2 is a newly identified member of the uncoupling protein family that shares high homology with UCP1. It was first cloned and characterized by Fleury et al. in 1997 [50]. The UCP2 gene is located on human chromosome 11q13 and mouse chromosome 7, and the protein consists of 309 amino acid residues with a relative molecular mass of approximately 32k. UCP2 shares 59% and 73% sequence homology with UCP1 and UCP3 [51], and it is a member of the mitochondrial anion carrier protein (MACP) family [52]. UCP2 works by dissipating the proton gradient across the mitochondrial inner membrane, leading to uncoupling of oxidative phosphorylation from ATP production. It plays a crucial role in regulating ATP and ROS generation [20], mitochondrial membrane potential (ΔΨm) [53], mitochondrial calcium homeostasis [54], immune response [37], cellular metabolism [31], and cell death [46]. Additionally, research has shown the involvement of UCP2 in various pathological conditions, including inflammation [37], metabolic disorders [55], cancer [56], and neurodegenerative diseases [57]. The roles it fulfills are immensely intricate (Table 1).

**Table 1:** Mitochondrial Protein UCP2 Plays a Key Role in Different Diseases (Partial Disease Overview)

Disease	UCP2 status	Impact	References
Melanoma	Overexpression of UCP2	Downregulation of glycolytic enzymes shifts glucose metabolism towards oxidative phosphorylation, resulting in decreased proliferation of B16F10 cells	[33]
Cholangiocarcinoma	UCP2^+/−^	Inhibition of glycolysis, thereby suppressing cell proliferation and growth	[58]
Colon tumor	UCP2^−/−^	Metabolic reprogramming and disruption of redox homeostasis contribute significantly to the exacerbation of CRC	[59]
Leukemia	Silencing of UCP2	HPB-ALL cellular metabolic redirection to glycolysis reduces cell proliferation in leukemia	[60]
Autoimmune encephalomyelitis	UCP2^−/−^	Increased inflammation	[61]
Leishmaniasis	UCP2^−/−^	Increasing ROS and pro-inflammatory cytokines to resist infection	[62]
Nonalcoholic fatty liver disease	Overexpression of UCP2	Alleviating hepatic oxidative stress and inflammatory responses to prevent the progression of liver injury, while compromising the liver’s ability to meet additional energy demands	[63]
Neurodegenerative diseases	Overexpression of UCP2	Neuroprotective effect	[57]

#### UCP2—Uncoupling or Transport of Metabolites?

**Fig. 1:**
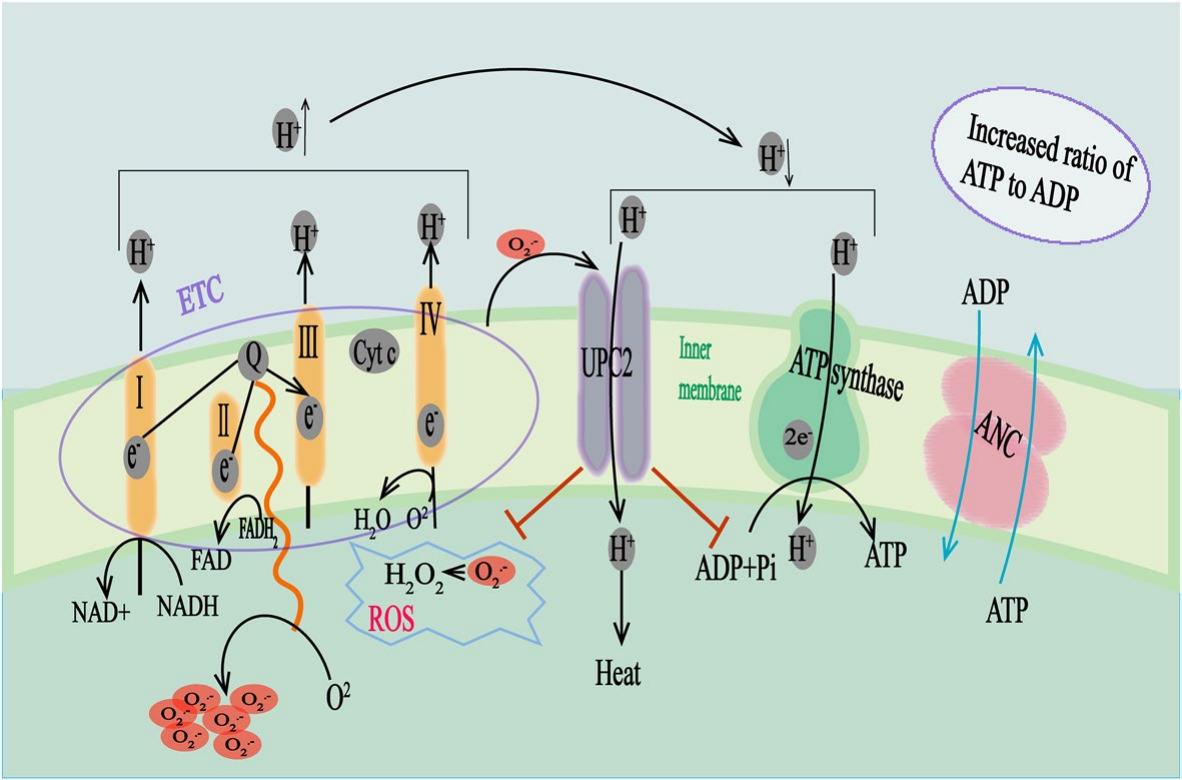
Schematic diagram of the electron transport chain (ETC), mitochondrial enzyme complexes, and UCP2 function. ADP adenosine diphosphate, ATP adenosine triphosphate, C4 4-carbon metabolite, ETC electron transport chain, FAD flavin adenine dinucleotide, H_2_O water molecule, H^+^ hydrogen ion, H_2_O_2_ hydrogen peroxide, NADH nicotinamide adenine dinucleotide, NADPH nicotinamide adenine dinucleotide phosphate, O^2−^ superoxide, Pi phosphate ion, ROS reactive oxygen species, UCP2 uncoupling protein 2, ANC adenine nucleotide translocase.

Based on the strong homology between UCP2 and UCP1, researchers initially believed that UCP2 possesses a similar mitochondrial membrane uncoupling function as UCP1. Under normal physiological conditions, UCP2 mediates proton leak across the mitochondrial inner membrane, allowing protons to directly enter the mitochondrial matrix. This process leads to a reduction in the proton electrochemical gradient on both sides of the membrane, causing a decoupling of the oxidative process from adenosine diphosphate (ADP) phosphorylation and resulting in a decrease in ATP synthesis. Consequently, the released energy is dissipated in the form of heat. Concomitantly, the reduction in the proton electrochemical gradient on both sides of the mitochondrial inner membrane also leads to a decrease in electron leakage in the respiratory chain, resulting in a reduction in the generation of reactive oxygen species (ROS) [64–66]. Additionally, UCP2 regulates the production of mitochondrial H_2_O_2_ [66] (Fig. 1).

Oxidative phosphorylation is a sophisticated process that produces high-energy ATP molecules through a series of chemical reactions facilitated by the electron transport chain (ETC). The ETC consists of complexes I–IV located on the inner membrane of mitochondria, along with ATP synthase. During this process, the flow of electrons begins with NADH (I) or FADH2 (II) at the electron carriers. Cytochrome oxidoreductase (III) and cytochrome c oxidase (IV) similarly pump protons through the membrane. Subsequently, the electrochemical gradient and proton flux across the inner membrane activate ATP synthase, which generates ATP through the reaction ADP + P_i_ + 3H ⇌ ATP + H^+^_2_O. Glucose metabolism is related to various biological processes within the cell. Under physiological conditions, when cellular energy demand is low, glucose metabolism plays a crucial role in various biological processes within the cell. Under normal physiological conditions, when cellular energy demand is low, UCP2 plays a role in dissociating the oxidation of substrates such as NADH, coenzyme Q, and cytochrome c from ADP phosphorylation, leading to a decrease in ATP synthesis and electrochemical membrane potential (ΔΨ). This enables UCP2 to lower ΔΨ levels, protecting against ROS-induced damage and cell death while dissipating the generated energy as heat.

**Fig. 2:**
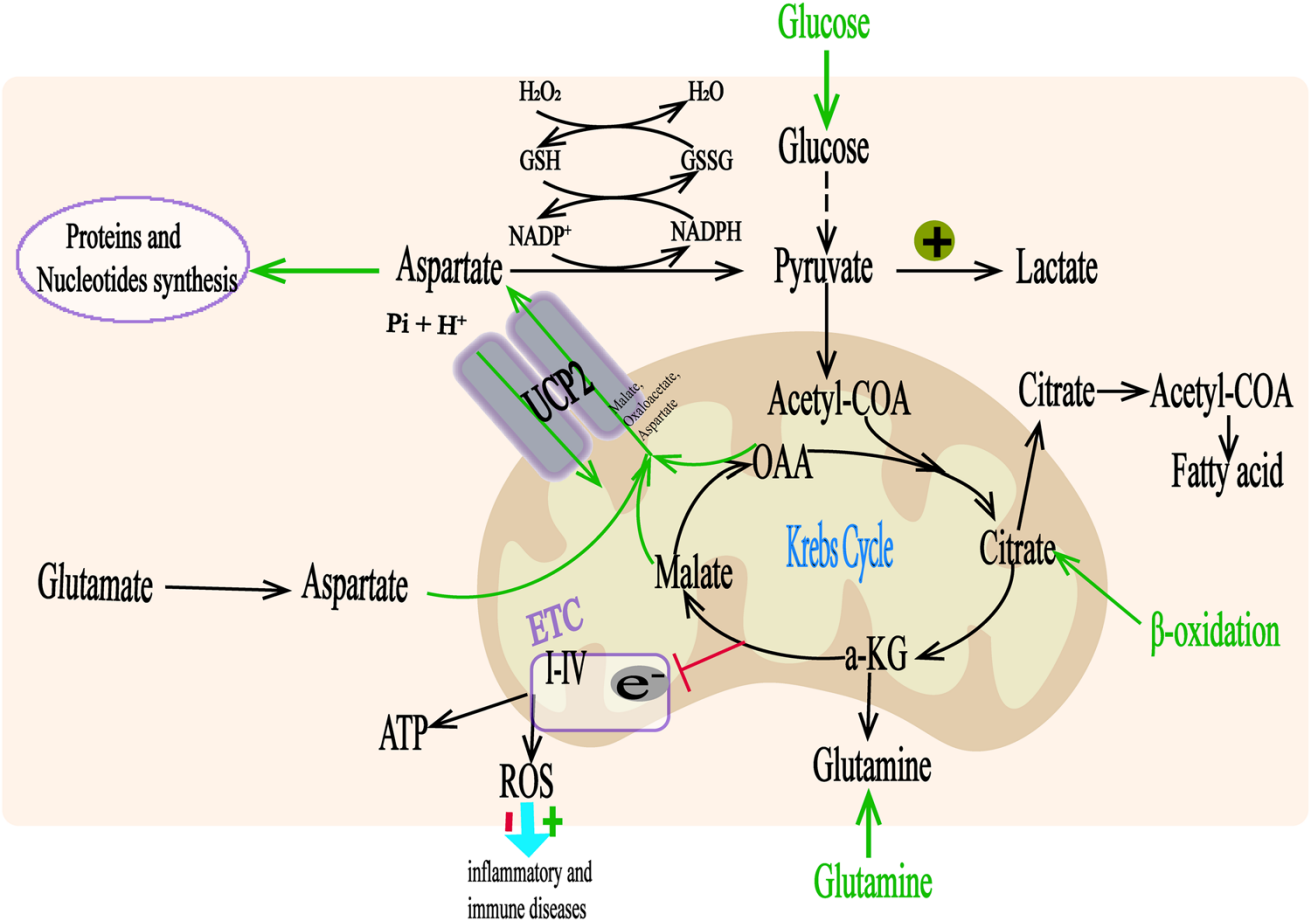
UCP2 plays a role in the transport of C4 metabolites. Ac-CoA acetyl-CoA, GSH glutathione, GSSG oxidized glutathione, OAA oxaloacetate, ROS reactive oxygen species, TCA tricarboxylic acid cycle, α-KG alpha-ketoglutarate.

Contrary to previous views, many scholars believe that the core role of UCP2 lies in the regulation of metabolic pathways rather than its uncoupling activity [67]. Experiments conducted by Pecqueur et al. [68] have demonstrated that there is no substantial difference in reactive oxygen species (ROS) levels between UCP2^+/+^ and UCP2^−/−^ cells. However, UCP2^−/−^ cells exhibited reduced fatty acid oxidation, increased glycolysis, and enhanced cell proliferation, indicating a heightened reliance on glycolysis. In contrast, UCP2 overexpression resulted in decreased proliferation and reduced reliance on glucose. These findings suggest that UCP2 regulates mitochondrial substrate utilization rather than uncoupling respiratory chain activity, thereby impacting ATP synthesis. Vozza et al. [69] experimentally confirmed this viewpoint in 2014 through metabolic and transport studies. This process can be specifically described as follows: UCP2 catalyzes the exchange of 4-carbon (C4) metabolites (such as malate, succinyl-CoA, and aspartate) from the tricarboxylic acid (TCA) cycle within the mitochondria with cytosolic phosphate ions through an H^+^-assisted mechanism. This mechanism is stimulated by the mitochondrial membrane potential and pH gradient. The export of C4 metabolites from mitochondria to the cytoplasm restricts the oxidation of pyruvate in the mitochondrial matrix, enhances the solubilization of glutamine, and increases the output of related C4 metabolites such as succinyl-CoA through the Krebs cycle, which is negatively controlled by it. This helps lower oxidative stress, ATP to ADP ratio, and ROS production in the mitochondrial respiratory chain. Additionally, UCP2 promotes the Warburg effect by redirecting glucose utilization towards lactate production. (Fig. 2).

### UCP2 Plays a Pivotal Role in the Pathogenesis of Sepsis

At the cellular and molecular levels, the pathogenesis of sepsis is intricately complex [70]. Within this intricate mechanism, UCP2 plays a crucial role as a pivotal mediator, exerting a wide range of biological effects contingent on its expression levels. Its potential as a therapeutic strategy for sepsis brings hope to patients afflicted by this condition. In a clinical study conducted by Jiang et al. [71], a total of 69 sepsis patients and 87 severely ill sepsis patients were selected, with 69 healthy volunteers serving as the control group. The levels of UCP2 in the blood cells of patients before and after treatment were detected by reverse transcription polymerase chain reaction and WB (Western blotting assay). The results revealed that both the mRNA and protein expressions of UCP2 were significantly higher in the blood cells of sepsis patients compared to healthy volunteers. This led the researchers to propose that UCP2 in blood cells could serve as a specific biomarker for sepsis, with its expression levels being positively associated with the severity of the disease.

With the advancement of experimental techniques, the overexpression of UCP2 has garnered increasing attention; Chen et al. [72] confirmed that UCP2 overexpression can significantly reverse the septic myocardial cell damage induced by LPS. In Geng et al.’s [73] study, Western blot results revealed increased expression of UCP2 in the CLP group compared to the Sham group. Fluorescence microscopy revealed that ROS production was significantly reduced in the UCP2 overexpression group compared to the CLP and AAV groups, and ELISA results suggested that lactate dehydrogenase (LDH), creatine kinase (CK), cardiac troponin I (cTnI), tumor necrosis factor-alpha (TNF-alpha), and interleukin-6 (IL-6) levels were significantly increased in the CLP and AAV groups compared to the Sham group. In contrast, the above cardiac enzymes and inflammatory cytokine secretion were significantly reduced in the UCP2 overexpression group compared with the CLP and AAV groups, confirming that UCP2 overexpression can suppress the generation of reactive oxygen species and inflammatory responses. This ultimately mitigates septic myocardial injury and reduces mortality rates [73].

## METABOLIC REPROGRAMMING DURING SEPSIS

**Fig. 3:**
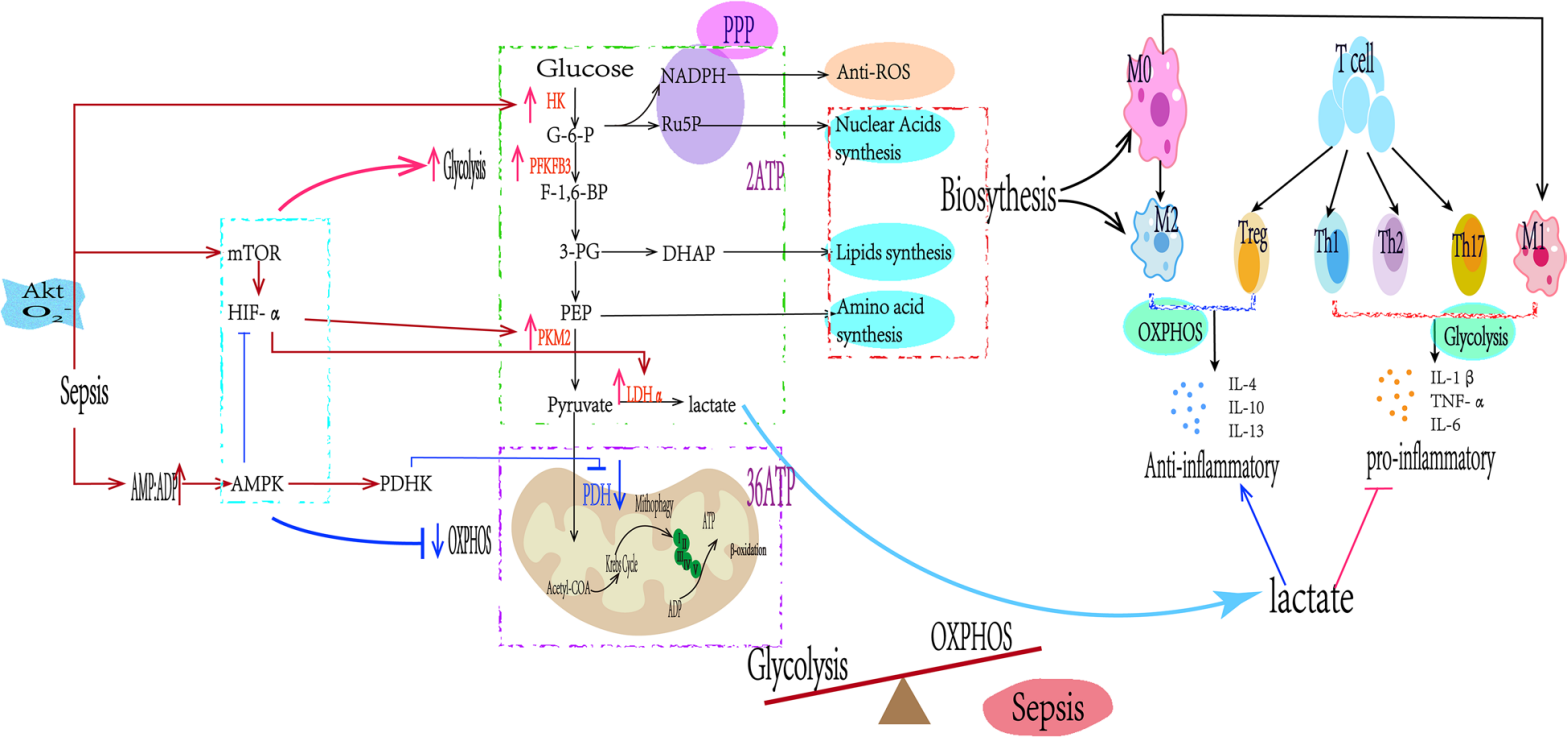
The intricate relationship between immunity and metabolism during sepsis.

Currently, the complexity of metabolism and inflammation processes has limited the development of effective therapeutic strategies for sepsis [74]. Sepsis causes dysfunction in both innate and adaptive immune responses, leading to excessive inflammation and immune suppression. Moreover, sepsis-induced mitochondrial damage and dysfunction contribute to cellular metabolic disturbances, reduced ATP synthesis, and oxidative stress, leading to apoptosis in organ cells and immune cells. This process is widely recognized as the primary underlying factor for immune dysregulation, multiple organ failure, and even mortality during sepsis [75]. However, the vital role of cellular metabolism in sepsis has been underestimated for a long time. During the hyperinflammatory phase, cells rely mainly on glycolysis rather than OXPHOS for energy production [26], a phenomenon known as the Warburg-like effect. Glycolysis is a necessary condition for the activation of host immune cells during the early stage of sepsis. However, lactate produced by aerobic glycolysis plays an immunosuppressive role [76] (Fig. 3). Understanding the impact of metabolism on immune cells is crucial for comprehending the imbalanced inflammatory response during sepsis. Shifting the metabolic profile of immune cells from glycolysis back to OXPHOS has been shown to facilitate the restoration of immune cell function during sepsis [77].

Akt protein kinase B; mTOR mechanistic target of rapamycin; HIF-α hypoxia-inducible factor alpha; AMPK AMP-activated protein kinase; PGC-1α peroxisome proliferator-activated receptor gamma coactivator 1-alpha; HK hexokinase; PFKB3 phosphofructokinase B-type; DHK dihydroxyacetone kinase; LDHα lactate dehydrogenase A; PDH pyruvate dehydrogenase; G-6-P glucose-6-phosphate; F-1,6-BP fructose-1,6-bisphosphate; 3-PG 3-phosphoglycerate; PEP phosphoenolpyruvate; DHAP dihydroxyacetone phosphate; PPP pentose phosphate pathway; NADPH nicotinamide adenine dinucleotide phosphate; Ru5P ribulose-5-phosphate; IL-1β interleukin-1 beta; TNF-α tumor necrosis factor alpha; IL-6 interleukin-6; IL-4 interleukin-4; IL-10 interleukin-10; IL-13 interleukin-13

M1 macrophages/Treg cells primarily utilize glycolysis for their metabolism, secreting pro-inflammatory cytokines such as IL-β, TNF-α, and IL-6. On the other hand, M2 macrophages and anti-inflammatory immune cells like Th1/Th2/Th17 secrete anti-inflammatory cytokines including IL-4, IL-10, and IL-13. In the hyperinflammatory phase of sepsis, cells predominantly utilize glycolysis as their primary metabolic pathway while suppressing oxidative phosphorylation (OXPHOS) in order to fulfill the cell’s growth requirements. Monocarboxylate transporters (MCTs) facilitate the transport of glycolytic metabolites, including lactate, out of the cell; lactate produced from glycolysis can promote an anti-inflammatory response, but high levels of lactate exert potent immunosuppressive effects on immune cells. The entire metabolic process is regulated by the mTOR/HIF-α and AMPK pathways, primarily through their impact on relevant metabolic enzymes.

### Excessive Inflammation and Metabolic Reprogramming

After microbial infection, pattern recognition receptors (PRRs) recognize pathogen-associated molecular patterns (PAMPs) or damage-associated molecular patterns (DAMPs) to activate immune cells, leading to the production of inflammatory responses [78, 79]. In the early stages of sepsis, abnormal activation of innate immune cells can trigger a severe inflammatory response. This leads to excessive release of inflammatory cytokines such as IL-1, TNF, and IL-17, resulting in a phenomenon known as “cytokine storm,” which causes damage to organs, tissues, and cells within the body [80] and has long been recognized as a significant contributor to high mortality rates during sepsis [81]. Excessive inflammation often results in energy deficiency, prompting immune cells to alter their metabolic profile, with glycolysis becoming their primary metabolic pathway [38]. This increases the availability of metabolic intermediates during the early stages of inflammation, providing sufficient energy for cell growth, differentiation, and immune responses [82], ensuring a rapid and effective immune response. Restricting glycolysis seems to alleviate inflammatory effects [83]. During this stage, the activity of enzymes such as 6-phosphofructo-2-kinase/fructose-2,6-bisphosphatase 3 (PFKFB3) [84] and pyruvate kinase M2 (PKM2) [85] is enhanced, promoting the conversion of glucose into lactate through glycolytic metabolism to accelerate energy production. This process is mainly regulated through the mTOR/HIF-α [86, 87] and AMPK [88] pathways. This process also enhances aerobic glycolysis and the pentose phosphate pathway to supply crucial precursor substances required for the rapid growth and proliferation of cells, including lipids, amino acids, and nucleotides [89]. Aerobic glycolysis plays an important role in the treatment of sepsis [85], and restricting the glycolytic pathway can also mitigate organ damage induced by the “inflammatory storm” stage [38]. In addition, Lu et al. [90] have demonstrated that inhibition of aerobic glycolysis can improve the prognosis of sepsis.

### Immune Suppression and Metabolic Reprogramming

Anti-inflammatory responses can alleviate tissue remodeling triggered by inflammatory reactions, thereby limiting local and systemic tissue damage [91]. Anti-inflammatory responses can alleviate tissue remodeling triggered by inflammatory reactions, thereby limiting local and systemic tissue damage. However, excessive anti-inflammatory responses may lead to immunoparalysis or compensatory anti-inflammatory response syndrome (CARS) [91], which can inhibit immune cell function and increase the risk of secondary infections. Some survivors of “cytokine storms” ultimately die during the immune suppression phase [22, 92]. The main features of immune suppression include increased expression of anti-inflammatory cytokines (such as IL-4, IL-10, and IL-37), recruitment of T cells, and immune cell death [23]. During the immune suppression phase, immune cells undergo a metabolic transition from glycolysis to fatty acid oxidation (FAO), which promotes an anti-inflammatory phenotype. During this stage, lactate, which is a metabolic byproduct of glycolysis, is transported out of cells through monocarboxylate transporters (MCT) [93]. Serum lactate levels significantly affect the prognosis of sepsis patients [94]. High levels of lactate promote death or inactivation of immune cells, leading to immune suppression and disruption of the body’s immune homeostasis [40]. Timely and effective regulation of glycolysis levels is also a focus of research in the immunosuppressive phase of sepsis, and inhibition of glycolysis levels may help to restore the function of immune cells [77], thereby positively impacting the prognosis of patients with sepsis.

## POTENTIAL THERAPEUTIC STRATEGY FOR SEPSIS—TARGETING UCP2 TO REGULATE GLUCOSE METABOLISM REPROGRAMMING

The relationship between metabolism and immunity during sepsis is extremely complex, with cell metabolic changes determining immune response [95]. UCP2, as a potential therapeutic target for sepsis, is not only widely expressed in immune cells but also involved in metabolic regulation. Its varied expression levels in sepsis are associated with different roles, and more researchers believe that overexpression of UCP2 plays a positive role in sepsis. However, the precise mechanisms underlying this protective effect remain to be fully elucidated.

### UCP2 and Metabolic Reprogramming

UCP2 facilitates the transport of C4 metabolites out of the mitochondria [69]. Its overexpression regulates glycolysis and the tricarboxylic acid (TCA) cycle [68]. Esteves et al. [96] demonstrated that UCP2 overexpression does not alter mitochondrial membrane potential or ATP synthesis. UCP2 overexpression alters the direction of metabolic substrate utilization by modulating the localization of metabolites involved in mitochondrial retrograde metabolism. Additionally, UCP2 overexpression leads to a decrease in lactate production under aerobic conditions. Instead, glucose is redirected towards the production of compounds such as alanine, oxaloacetate, and acetyl-CoA, resulting in a higher proportion of carbon entering the TCA cycle. This elevates the levels of α-ketoglutarate (α-KG), succinate, fumarate, and malate within the TCA cycle [97]. Currently, the therapeutic strategy of using UCP2 to regulate metabolic reprogramming in tumors has been confirmed in cancer research. The study conducted by Esteves et al. [33] has confirmed that UCP2 regulates the metabolic shift from glycolysis to oxidative phosphorylation, leading to a significant decrease in the proliferation of B16F10 cells and consequently reducing their tumorigenic capacity.

### Targeting UCP2 for Regulating Glucose Metabolism Reprogramming Impacts Sepsis

UCP2 serves as a key factor in the metabolic regulation of sepsis. Silencing UCP2 further intensifies the Warburg effect, while overexpression of UCP2 restricts glycolysis and inhibits the expression of oxidases, thereby alleviating the damage caused by sepsis. Ding et al. [34] confirmed that UCP2 overexpression protects against endotoxin-induced HK-2 cell damage. This protection is achieved through the inhibition of apoptosis, inflammation, oxidative stress, matrix metalloproteinase loss, and reactive oxygen species (ROS) production. Overexpression of UCP2 also increases ATP production and mitochondrial DNA content while improving mitochondrial ultrastructure damage. On the other hand, downregulation of UCP2 exacerbates endotoxin-induced kidney damage, inflammation, macrophage infiltration, mitochondrial dysfunction, and oxidative stress. Ji et al. [43] found that the expression of UCP2 was significantly increased in renal tissues of mice with infectious AKI due to CLP treatment and in renal tubular epithelial (HK-2) cells induced by LPS (5 μg/mL) treatment for 24 h. There were an upregulation of PEP, pyruvic acid, and lactic acid and a downregulation of glucose in the two groups of the sepsis model. Increased lactate during this process exacerbated mitochondrial damage in HK-2 cells, and it was further demonstrated experimentally that upregulated UCP2 attenuated the Warburg effect and alleviated LPS-induced mitochondrial dysfunction in renal tubular epithelial cells.

## SUMMARY AND DISCUSSION

The pathogenesis of sepsis is extremely complex [98], and metabolic dysfunction coupled with immune paralysis leading to late mortality remains a major challenge in clinical practice [99]. With the advancements in metabolism and immunology, metabolic reprogramming has come into focus. It has been discovered that cells primarily rely on mitochondrial-driven oxidative phosphorylation (OXPHOS) during resting state [100]. During the hyperinflammatory phase of sepsis, immune cells undergo a rapid metabolic shift due to energy deficiency, with glycolysis becoming the primary metabolic pathway while OXPHOS is inhibited [26], to provide ATP and metabolites for cellular activity in the early phase of sepsis [101]. Moreover, it is important to note that the production of lactate through glycolysis suppresses immune responses [102]. The intricate interplay between immunity and metabolism poses limitations on sepsis treatment [83]. Mitochondria, as the powerhouse of cellular energy metabolism, play a crucial role in the development of sepsis [103]. Pecqueur et al. [104] proposed that UCP2 located on the inner mitochondrial membrane may serve as a mediator for coupling between glucose oxidation and mitochondrial metabolism. UCP2 is widely distributed in various tissues and participates in immune and metabolic processes. UCP2 has been regarded as a critical mediator in the pathogenesis of sepsis, and its varying expression levels play a distinct role in the pathogenesis of this condition.

In sepsis-induced astrocytes, studies have revealed an increase in UCP2 protein levels and a decrease in mitochondrial membrane potential (MMP) and ATP levels, as well as mitochondrial damage. It has been observed that silencing UCP2 amplifies the expression of pro-inflammatory markers and exacerbates mitochondrial ultrastructural damage. These findings suggest that UCP2 might play a protective role in septic conditions [42]. More and more scholars believe that overexpression of UCP2 can reverse the damage caused by sepsis; this study started from metabolomics and found that UCP2 overexpression is closely related to metabolic reprogramming. That is, unlike the increased expression of glycolysis and mitochondrial damage observed after UCP2 silencing, UCP2 overexpression generates retrograde signaling in mitochondria, changes the expression of glycolysis and oxidative enzymes, and enhances oxidative phosphorylation [33], which reduces the expression of inflammatory factors and ameliorates mitochondrial damage, while restoring OXPHOS function to the reduction of sepsis mortality [105]. The above-mentioned research confirms the potential therapeutic strategy of UCP2 overexpression for sepsis, and the realization of this treatment mechanism may rely on the regulation of UCP2 on the intermediate link of glycolysis.

In summary, UCP2, one of the key targets in sepsis, is involved in the complex metabolic relationship between excessive inflammation and immunosuppression. UCP2 overexpression regulates the reprogramming of immune cell metabolism, effectively inhibits organ damage caused by excessive inflammation, prevents immune paralysis, improves patient outcomes, and addresses the challenging issues of sepsis that arise from immune and metabolic dysregulation, ultimately enhancing the survival rate of sepsis patients. These findings provide novel and valuable insights for the treatment and prognosis of sepsis. Although scientists’ understanding of the pathogenesis of sepsis has improved dramatically, through the use of “omics” analysis techniques that can simultaneously analyze multiple levels of RNA, proteins, lipids, and metabolites [106], revealing the complexity of sepsis immune response and inflammation, more efforts are needed to translate these new discoveries into effective therapeutic strategies. This study offers a comprehensive overview of the potential application of UCP2 in the treatment of sepsis and outlines future research directions. That is, the study by Ji et al. [43] suggests the construction of a stable CLP model, which can accurately simulate the pathophysiological processes of sepsis, making it suitable for in-depth investigation into the regulatory roles of UCP2, utilizing LV5-UCP2 lentiviral vectors and si-UCP2 small interfering RNAs to modulate UCP2 expression, either by upregulation or downregulation. Inflammatory responses, metabolic enzymes, related metabolites (such as hexokinase and lactate), oxidative stress, cell damage, and survival rates within the sepsis model were observed, and molecular and cellular biology techniques such as Western blotting, real-time quantitative PCR, and flow cytometry were employed to delve into the molecular mechanisms by which UCP2 modulates sepsis. This thorough methodology will deepen insights into the role of UCP2 in the management of sepsis and provide a scientific foundation for the discovery of new therapeutic drugs.

## Data Availability

No datasets were generated or analyzed during the current study.
